# Interfacial Shear Fracture Behavior of C18150Cu/1060Al/C18150Cu Trilayered Composite at Different Temperatures

**DOI:** 10.3390/ma18030559

**Published:** 2025-01-26

**Authors:** Huisheng Cai, Siqi Yang, Qudong Wang, Yuchao Zhao, Qixiang Jia, Mahmoud Ebrahimi, Liang Liu, Feng Guo, Zhengping Shang

**Affiliations:** 1 National Engineering Research Center of Light Alloy Net Forming and State Key Laboratory of Metal Matrix Composites, School of Materials Science and Engineering, Shanghai Jiao Tong University, Shanghai 200240, China; caihuisheng@imut.edu.cn (H.C.); zhaoyuchao@sjtu.edu.cn (Y.Z.); ebrahimi@maragheh.ac.ir (M.E.); 2 Inner Mongolia Key Laboratory of New Materials and Surface Engineering, School of Material Science and Engineering, Inner Mongolia University of Technology, Hohhot 010051, China; 20241800329@imut.edu.cn (S.Y.); fly_liuliang@imut.edu.cn (L.L.); guofengnmg@sina.com (F.G.); 3 Department of Mechanical Engineering, Faculty of Engineering, University of Maragheh, Maragheh 83111-55181, Iran; 4 Jiangsu Zhongse Composite Materials Co., Ltd., Wuxi 214000, China; ly_szp@163.com

**Keywords:** C18150Cu/1060Al/C18150Cu trilayered composites, interfacial intermetallics, shear strength, shear fracture mechanism, temperature

## Abstract

Interfacial shear fracture behavior of C18150Cu/1060Al/C18150Cu trilayered composite at different temperatures, which was fabricated by high-temperature oxygen-free hot rolling technology. The interfacial microstructure, interfacial shear strength, interfacial shear fracture morphology, and microstructure near the shear fracture were systematically investigated. The results reveal that the composite exhibits a metallurgical and mechanical bonding interface, along with mechanical interlocking between the copper and aluminum. As the testing temperature increases, the interfacial shear strength decreases. At temperatures below 150 °C, the strength remains stable, but it sharply decreases at temperatures above 150 °C. Specifically, the interfacial shear strength is 56.8 MPa at room temperature and 20.9 MPa at 350 °C. When the testing temperature is below 100 °C, the interfacial shear fracture predominantly occurs at the interface between the copper alloy and intermetallics. Also, aluminum is attached to the copper surface of the shear fracture, and the size and quantity of attached aluminum increase with the increase in temperature. When the testing temperature exceeds 100 °C, curled aluminum appears on the copper layer, and a large number of intermetallics are attached to the aluminum surface. This indicates that the bonding strength between intermetallics and aluminum is higher than that between intermetallics and copper.

## 1. Introduction

The advantages of copper/aluminum layered composites, such as their excellent electrical and thermal conductivity, lightweight, and low cost, offer broad market application prospects in the fields of electrical and electronics, new energy vehicles, and mobile communications. Copper/aluminum layered composites have effectively replaced copper or copper alloys in battery current collectors, vehicle secondary batteries, positive and negative conversion components of electric vehicles, LED lighting, building decoration, and other fields [1,2,3].

Currently, copper/aluminum layered composites are mainly prepared by hot rolling [4], cold rolling [5], cast rolling [6], accumulative roll bonding (ARB) [7], pulsed-current-assisted vacuum sintering processes [8], corrugated + flat rolling (CFR) [9], rotary swaging [10,11,12,13], axi-symmetric forward spiral composite extrusion (AFSCE) [14], high-pressure torsion (HPT) [15], and other composite technologies. In addition, in order to achieve better composite effects, the combination of various composite technologies to prepare layered composites has also attracted increasing attention, such as cold spray combined with laser melting [16], accumulative roll bonding (ARB) with cold roll bonding (CRB) [17], diffusion bonding with conventional rolling [18], etc.

Considering the plastic deformation of copper and aluminum during the composite process, as well as their synergistic deformation ability, pure copper and pure aluminum or aluminum alloys with a lower content of alloying elements are usually selected to prepare copper/aluminum layered composites [19,20,21]. Numerous studies have focused on the fabrication of layered composites using pure copper and pure aluminum. The interfacial intermetallics (IMCs) of copper/aluminum layered composites are formed by diffusion. The interfacial IMCs of copper/aluminum layered composites are mainly composed of Al_2_Cu, AlCu, Al_3_Cu_4_, Al_3_Cu_2_, Al_4_Cu_9_, and other intermetallics [22,23,24,25]. Through the formation of interfacial IMCs, the metallurgical bonding interface is formed, which can effectively improve the interfacial shear strength of the composites. However, the mechanical properties of pure copper and pure aluminum are relatively poor, and the tensile properties of copper/aluminum layered composites prepared from these materials are typically also weak.

In order to improve the comprehensive mechanical properties of copper/aluminum layered composites, it is an effective method to select copper alloy with high strength and toughness to prepare copper/aluminum layered composites [26]. The C18150 copper alloy, a type of Cu-Cr-Zr copper alloy, possesses high strength and conductivity, resulting in excellent mechanical properties and electrical conductivity [27,28]. The copper/aluminum layered composites prepared by the C18150 copper alloy can improve their tensile properties under the premise of ensuring good electrical and thermal conductivity. Nonetheless, the interfacial shear strength remains a critical performance metric for assessing the effectiveness of the bonding [29,30]. In addition, the actual working conditions of copper/aluminum layered composites often involve various temperature environments. In this regard, the interfacial shear properties and shear fracture mechanism of copper/aluminum layered composites prepared by C18150 copper alloy and aluminum at different temperatures need to be systematically studied and analyzed. A comprehensive study of the interfacial shear properties and fracture mechanisms at various temperatures will aid in optimizing processing parameters and allow for more precise control over the interfacial microstructure of the composites. This approach will provide valuable theoretical insights into enhancing the interfacial bonding strength, which in turn will contribute to improving the overall performance and reliability of copper/aluminum layered composites.

In this study, we prepared the C18150Cu/1060Al/C18150Cu trilayered composites using C18150 copper alloy and 1060 aluminum alloy. We investigated the interfacial shear strength of the composites at various testing temperatures, as well as the types of cracks that occurred at those temperatures. Finally, we clarified the interfacial shear fracture mechanism of the composites.

## 2. Materials and Methods

C18150Cu/1060Al/C18150Cu trilayered composites were prepared using 1060 aluminum alloy strip and C18150 copper alloy strip. The chemical compositions of 1060 aluminum alloy and C18150 copper alloy are presented in Table 1 and Table 2, respectively.

**Figure 1:**
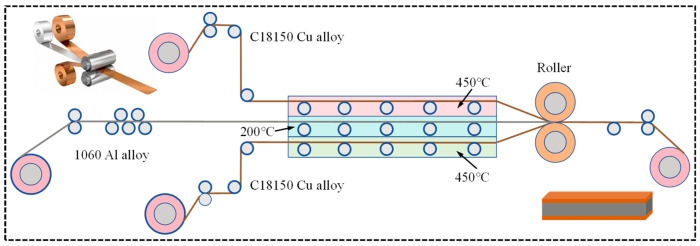
The schematic representation of the preparation process of C18150Cu/1060Al/C18150Cu trilayered composites by high-temperature oxygen-free hot rolling [31].

High-temperature, oxygen-free hot rolling technology was employed to produce C18150Cu/1060Al/C18150Cu trilayered composites. This was carried out in order to minimize the impact of interfacial oxidation on the bonding strength during the composite fabrication process. The schematic representation of the mentioned composite preparation is shown in Figure 1. Initially, C18150 copper alloy and 1060 aluminum alloy were preheated in a three-layer independent holding furnace at 450 and 200 °C, respectively. The preheating atmosphere was a reducing atmosphere (A mixed gas composed of 25% N_2_ and 75% H_2_). Following preheating, C18150 copper alloy, 1060 aluminum alloy, and C18150 copper alloy were prepared into trilayered composites by a rolling mill. In order to better analyze the influence of interfacial microstructures on the interfacial shear strength of the composites, the interfacial microstructures parallel to the rolling direction and perpendicular to the rolling direction were analyzed.

**Figure 2:**
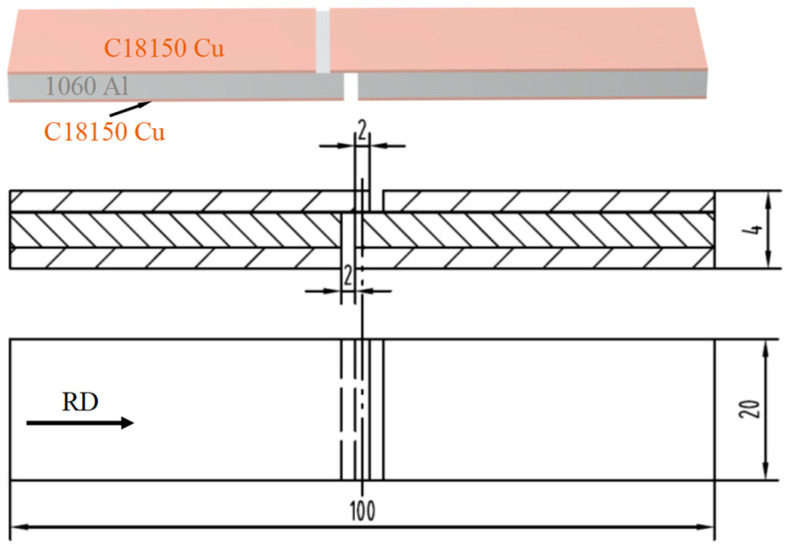
The size of the shear testing sample of C18150Cu/1060Al/C18150Cu trilayered composite [31].

The FEI-Apreo 2S HiVac scanning electron microscope (SEM, Thermo Fisher Scietific, Waltham, MA, USA) was utilized to study the interfacial microstructure of the C18150Cu/1060Al/C18150Cu trilayered composite both parallel and perpendicular to the rolling direction. Also, an energy-dispersive spectrometer (EDS, Shimadzu, Kyoto, Japan) was applied to find out the chemical composition of the interfacial microstructure. The interfacial shear strength of the composites at 25, 100, 150, 200, 250, 300, and 350 °C was examined by the WDW-100G high-temperature electronic universal testing machine (Jilin Guanteng Automation Technology Co., Ltd., Jilin, China) at the tensile speed of 0.2 mm/min. Three tests were carried out at each temperature, and the average value was taken as the interfacial shear strength of the composites. In this regard, the size of the shear testing sample is shown in Figure 2. In addition, the SEM was used to investigate the shear fracture and the microstructure near the shear fracture of the composites. An energy-dispersive spectrometer was used to study the element distribution of the shear fracture and the microstructure near the shear fracture in order to find out how the composite’s interfacial shear fracture happened.

## 3. Results and Discussion

### 3.1. Interfacial Microstructure

**Figure 3:**
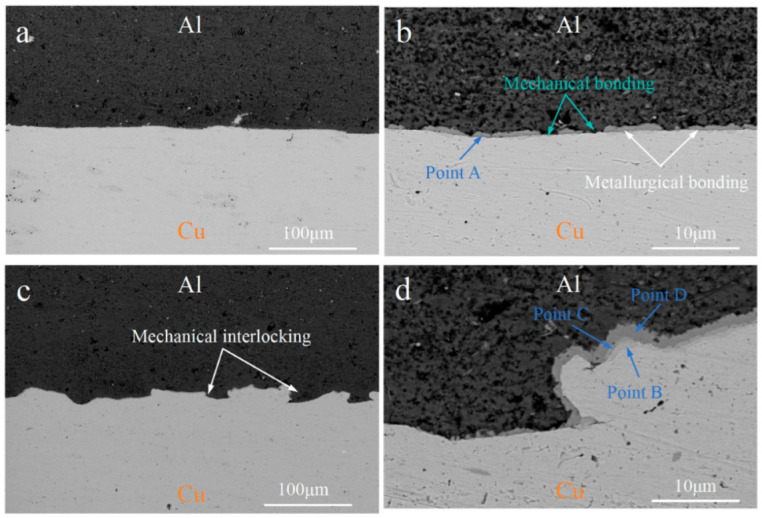
The interfacial microstructure of the C18150Cu/1060Al/C18150Cu trilayered composite: (**a**,**b**) parallel to the rolling direction and (**c**,**d**) perpendicular to the rolling direction.

The interfacial microstructure and EDS of the C18150Cu/1060Al/C18150Cu trilayered composites, both parallel and perpendicular to the rolling direction, are presented in Figure 3 and Table 3, respectively. As can be observed, a distinct thickness of interfacial intermetallic compounds (IMCs) forms between the copper and aluminum layers. The distribution of these interfacial IMCs is fairly random. It means that the interface bonding of the prepared composites consists of the metallurgical bonding (about 80%) with the IMCs and the mechanical bonding (about 20%) without IMCs. The formation of metallurgical bonding interface is the result of the mutual diffusion of copper and aluminum elements and the formation of intermetallic compounds through solid-state phase transition. The EDS results for point A indicate that the thinner interfacial IMCs have a diffraction contrast that is Al_2_Cu. As the thickness of the IMCs increases, three distinct diffraction patterns emerge, corresponding to the IMCs Al_2_Cu, AlCu, and Al_4_Cu_9_. Specifically, the intermetallic compounds near the copper side are primarily Al_4_Cu_9_, while those near the aluminum side are Al_2_Cu, with the intermediate layer consisting of AlCu.

Specifically, the intermetallic compounds near the copper side are primarily Al4Cu9, while those near the aluminum side are Al_2_Cu, with the intermediate layer consisting of AlCu. In general, Al_2_Cu, AlCu, and Al_4_Cu_9_ are observed at the interface of copper/aluminum layered composites prepared by solid–liquid composite technology or after annealing heat treatment [32,33,34]. Additionally, the interface of the composites, whether parallel or perpendicular to the rolling direction, exhibits distinct characteristics. Accordingly, the interface parallel to the rolling direction is mostly flat, while the interface perpendicular to the rolling direction presents a large number of positions where copper and aluminum are meshing with each other, thus forming a mechanical interlocking between copper and aluminum.

### 3.2. Interfacial Shear Strength of Composite

**Figure 4:**
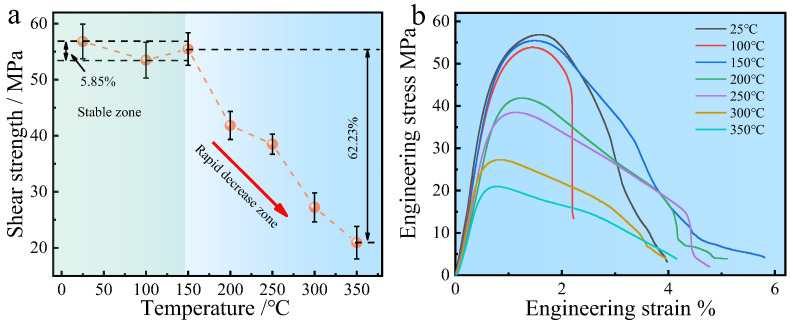
The interfacial shear strength and stress–strain curves of the C18150Cu/1060Al/C18150Cu trilayered composite at different testing temperatures: (**a**) the relationship between interfacial shear strength and testing temperature and (**b**) stress–strain curves of the composites.

Figure 4 represents the stress–strain curves and the corresponding shear strengths of the C18150Cu/1060Al/C18150Cu trilayered composites at different temperatures of 25, 100, 150, 200, 250, 300, and 350 °C. At room temperature, the interfacial shear strength of the composites is 56.8 MPa. The interfacial shear strength generally shows a decreasing trend with the increase in the testing temperature. However, the interfacial shear strength of the composites can be maintained above 50 MPa as the temperature is less than 150 °C. Indeed, the temperature does not change the shear strength much in this temperature range; the range of changes in shear strength is only 6%. This means that the interfacial shear strength is very stable at temperatures below 150 °C. Once the temperature exceeds 150 °C, the interfacial shear strength decreases sharply. At 350 °C, the interfacial shear strength drops to 20.9 MPa, representing a decrease of approximately 62%. Table 4 displays the interfacial shear strength of the C18150Cu/1060Al/C18150Cu trilayered composite. The interfacial shear strength of composites prepared by high-temperature oxygen-free hot rolling is significantly higher than that of composites prepared by other preparation methods, 45 MPa [35], 32 MPa [36], 36 MPa [37], 27 MPa [38], 26 MPa [39].

In our previous study [31], it was found that the interfacial shear strength of Cu/Al/Cu trilayered composites made from pure copper and 1060 aluminum alloy drops quickly as the testing temperature increases. The interface, which consists of both metallurgical and mechanical bondings, also forms IMCs with a discontinuous distribution. However, there is a mechanical interlocking between copper and aluminum at the interface of the composites made from the C18150Cu and the 1060Al, in addition to the metallurgical and mechanical bonds. In this regard, the high-temperature strength of the C18150 copper alloy surpasses that of pure copper, resulting in a significantly smaller strength decrease as temperature increases compared to pure copper. Therefore, when the testing temperature increases, the mechanical interlocking at the interface maintains the interfacial shear strength. Indeed, the mechanical interlocking between the copper and aluminum at the interface of the composites is a significant reason why the interfacial shear strength of the mentioned composites remains stable as the temperature is below 150 °C.

### 3.3. Interfacial Shear Fractography of Composite

**Figure 5:**
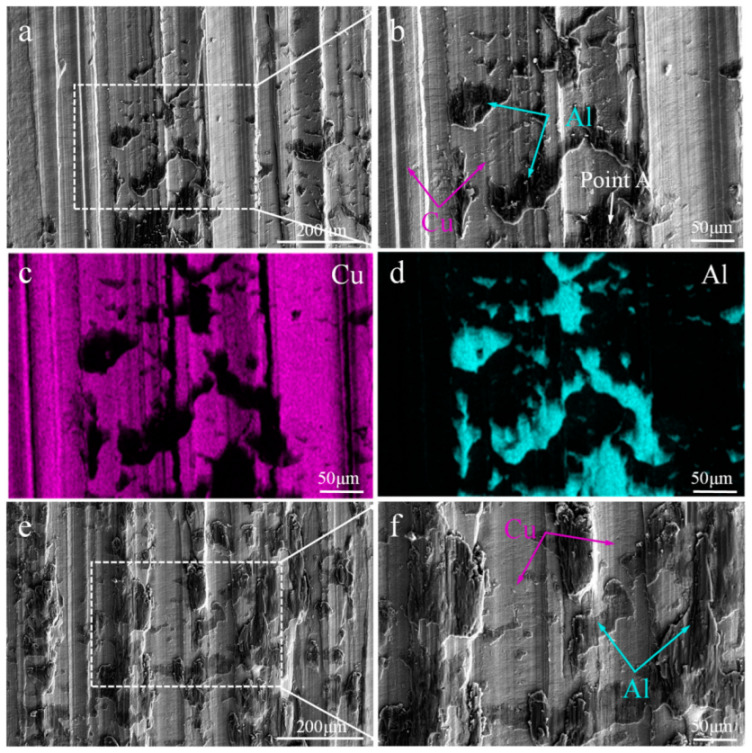
The shear fracture of the C18150Cu/1060Al/C18150Cu trilayered composite: (**a**,**b**) the shear fracture at room temperature; (**c**,**d**) EDS mapping of shear fracture tested at room temperature; (**e**,**f**) the shear fracture tested at 100 °C.

The shear fracture of the C18150Cu/1060Al/C18150Cu trilayered composites at room temperature and 100 °C is shown in Figure 5. It can be observed that the shear fracture predominantly occurs at the composite interface while a small amount of irregularly shaped aluminum is attached to the copper surface of the shear fracture. The EDS of point A shows that the chemical composition is Al (99.16 at.%, 98.04 wt.%) and Cu (0.84 at.%, 1.96 wt.%). Furthermore, the EDS mapping results reveal that, at room temperature, the shear fracture predominantly exposes copper, with only a few regions showing aluminum attached to the copper surface. This indicates that there are no IMCs on the copper shear fracture surface. In this regard, the interfacial IMCs mainly exist on the aluminum surface of the shear fracture, while only a small amount of IMCs exist on the copper surface of the shear fracture. Therefore, the interfacial bonding strength between IMCs and aluminum is higher than that between IMCs and copper.

The shear fracture of the composites at 100 °C also mainly occurs at the interface, and some unpeeled aluminum is attached to the copper surface. However, compared with the shear fracture at room temperature, more unpeeled aluminum is attached to the copper surface of the shear fracture at 100 °C. This is mainly due to the fact that the strength of aluminum decreases with the increase in temperature, which leads to the actual interfacial bonding strength at more and more locations being higher than the strength of the aluminum at 100 °C. Consequently, at some locations, the aluminum layer fails and fractures without causing a shear fracture at the interface. In general, shear fractures in the composites occur at the interface when the temperature is between room temperature and 100 °C, with aluminum attaching to the copper surface at the fracture.

**Figure 6:**
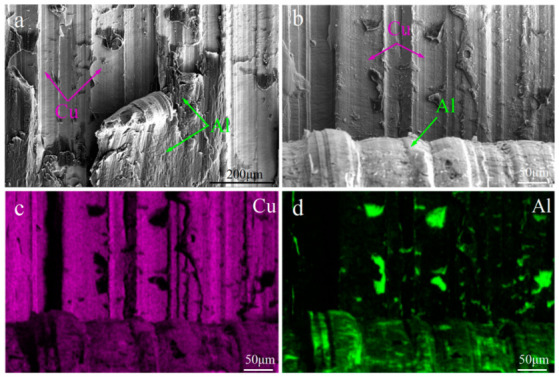
The shear fracture of the C18150Cu/1060Al/C18150Cu trilayered composite tested at 150 °C: (**a**,**b**) the shear fracture tested at 150 °C and (**c**,**d**) EDS mapping of shear fracture tested at 150 °C.

The shear fracture of C18150Cu/1060Al/C18150Cu trilayered composites at 150 °C is shown in Figure 6. Accordingly, the shear fracture mainly occurs at the interface. Also, some aluminum is attached to the copper surface. However, large flakes of unpeeled aluminum begin to appear on the fracture, simultaneously, as shown in Figure 6a,b. The strength of aluminum decreases sharply with temperature increases. With the peeling of aluminum and copper, when the strength of aluminum is lower than the interfacial bonding strength, the fracture of aluminum occurs, thus the large, flake-like pieces of aluminum that are not completely peeled off from copper appear at the interface. The unpeeled aluminum is curled along the tensile direction. Simultaneously, the EDS mapping results reveal the presence of copper element on the unpeeled aluminum surface. However, the copper surface of the shear fracture, except for the attached aluminum, exhibits almost no aluminum distribution, indicating that the IMCs exist on the aluminum surface of the shear fracture. These experimental results further confirm that the bonding strength between IMCs and aluminum is greater than the bonding strength between IMCs and copper.

**Figure 7:**
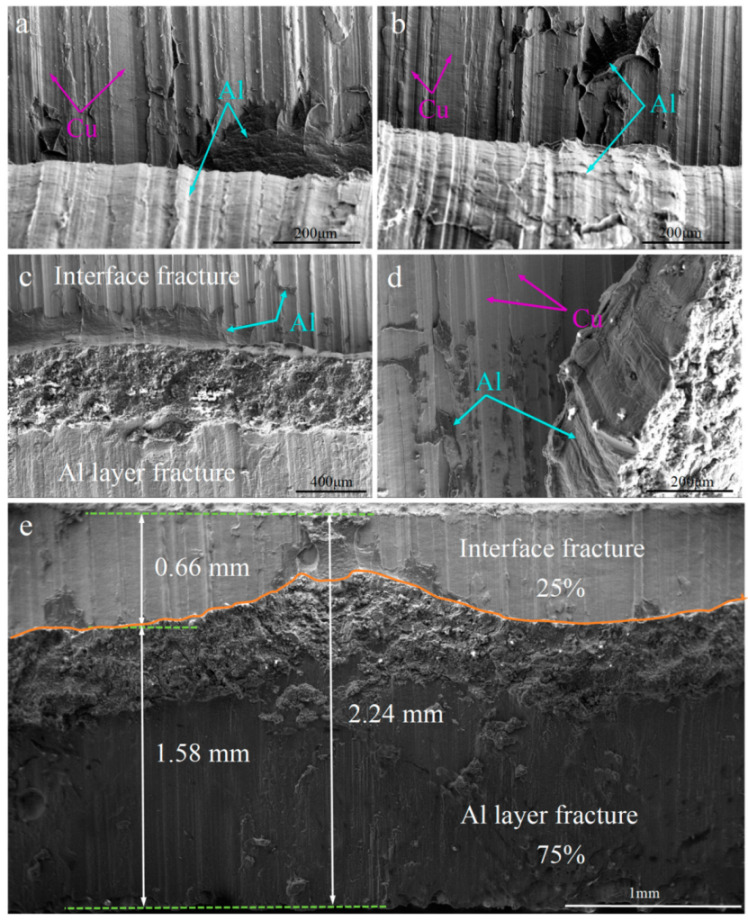
The shear fracture of the C18150Cu/1060Al/C18150Cu trilayered composite tested at different temperatures of (**a**) 200 °C; (**b**) 250 °C; (**c**,**d**) 300 °C; (**e**) 350 °C.

Figure 7 represents the shear fracture of the C18150Cu/1060Al/C18150Cu trilayered composite at 200, 250, 300, and 350 °C. Accordingly, the shear fracture is similar to the above fracture mode. During the shear fracture, the curled aluminum is not fully peeled off the copper, and the irregularly shaped aluminum is still attached to the copper surface. As the temperature increases, the amount of curled and incompletely peeled aluminum at the shear fracture also increases. When the shear test was carried out at 300 °C, the shear fracture showed obvious signs of aluminum layer fracture, as shown in Figure 7c. The shear fracture at a testing temperature of 350 °C basically includes the aluminum layer fracture. At this time, the strength of aluminum is the lowest in the experimental temperature range, and the gap between the aluminum strength and interfacial shear strength is the largest. It can be observed from Figure 7e that when the shear fracture occurs at 350 °C, the fracture of the aluminum layer reaches nearly 75%, and the fracture at the interface is about 25%. This issue also demonstrates that an increase in testing temperature leads to a sharp decrease in the strength of the aluminum layer, which gradually falls below the interface bonding strength. Consequently, more and more aluminum is attached to the copper surface until the fracture of the aluminum layer occurs.

### 3.4. Microstructure near the Shear Fracture of Composite

**Figure 8:**
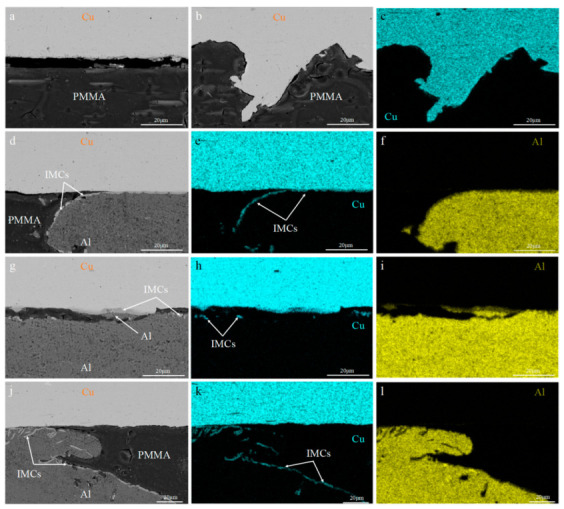
The microstructure near the shear fracture of the C18150Cu/1060Al/C18150Cu trilayered composite tested at different temperatures of (**a**) 25 °C; (**b**,**c**) 100 °C; (**d**–**f**) 200 °C; (**g**–**i**) 350 °C; (**j**–**l**) 350 °C.

Figure 8 shows the microstructure of the C18150Cu/1060Al/C18150Cu trilayered composite near the shear fracture at different temperatures. When the shear test was carried out at room temperature and 100 °C, the shear fracture mainly occurred at the interface. Figure 8a–c shows that there are no attached IMCs on the copper surface, and the copper and aluminum broke in a way that meshes with each other. As the testing temperature increases, the shear fracture reveals a curled aluminum layer that remains partially peeled off from the copper. The presence of IMCs on the unpeeled aluminum surface is easily visible. This further confirms that the bonding strength of IMCs with aluminum is higher than the bonding strength of IMCs with copper, as seen in Figure 8d–f. Simultaneously, the aluminum attached to the copper surface can also be observed in the microstructure near the shear fracture, which is consistent with the shear fracture observation results. The distribution and accumulation of IMCs in the aluminum layer do not completely peel off the copper when the shear test was performed at 350 °C. The strength of aluminum is very poor at 350 °C, so the hard and brittle IMCs are continuously subjected to shear, extrusion, and movement in the low-strength aluminum layer during the shear fracture process, so that the accumulation phenomenon occurs in the aluminum layer.

### 3.5. Interfacial Shear Fracture Mechanism of Composite

**Figure 9:**
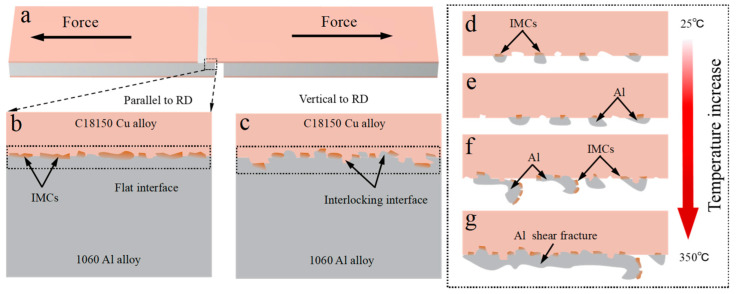
The schematic representation of the interfacial shear mechanism of the C18150Cu/1060Al/C18150Cu trilayered composite prepared by high-temperature oxygen-free rolling at different temperatures of (**a**) shear testing sample; (**b**) the interfacial microstructure parallel to the rolling direction; (**c**) the interfacial microstructure perpendicular to the rolling direction and (**d**–**g**) the interfacial shear fracture at different temperature.

As previously mentioned, the interfacial microstructure of the C18150Cu/1060Al/C18150Cu trilayered composites fabricated by high-temperature oxygen-free rolling exhibits the following features. The parallel interface to the rolling direction, which is relatively flat and forms IMCs with a discontinuous distribution. In addition to the formation of IMCs with a discontinuous distribution at the interface perpendicular to the rolling direction, there is also mutual meshing between copper and aluminum, which results in the formation of a mechanical interlocking. This indicates that copper and aluminum undergo different degrees of plastic deformation along the rolling direction during the rolling bonding process. The composites can achieve a better interfacial shear strength as they mechanically interlock, see Figure 9b,c.

The shear fracture of the composites showed various fracture morphologies after shear testing at various temperatures. The shear fracture of the composites mainly occurs at the interface at room temperature. A small amount of small-sized aluminum is attached to the copper surface at the shear fracture. Also, there are IMCs at the interface between the attached aluminum and copper. With the increase in the testing temperature, the size and content of the attached aluminum to the copper surface are gradually increased, but there are almost no IMCs on the copper surface. At low temperatures, shear fracture of composites mostly occurs at the interface, and the interfacial bonding strength between the interface and the material is not as strong as aluminum. However, with the increase in the testing temperature, the strength of the aluminum decreases sharply, resulting in the increase in the attached aluminum to the copper surface at the shear fracture, until the emergence of large, flake-like aluminum and curled aluminum that is not completely peeled off from the copper, and the IMCs are attached to the unpeeled aluminum surface. When the testing temperature is further increased, the shear fracture of the composites is transformed into the aluminum layer fracture, along with a small amount of interface fracture, until the complete aluminum layer fracture occurs. It is worth noting that when the shear fracture occurs at the interface, the interface is actually the interface between the IMCs and copper. It means that the bonding strength between the IMCs and copper is lower than the bonding strength between the IMCs and aluminum. The interface shear mechanism of the prepared C18150Cu/1060Al/C18150Cu trilayered composite by high-temperature oxygen-free rolling at different temperatures is shown in Figure 9d–g.

The testing temperature plays a crucial role in determining the shear fracture mechanism of the composites. As the testing temperature increases, the shear fracture gradually transitions from an interface shear fracture to a mixed shear fracture, where both the aluminum shear fracture and the interface shear fracture occur simultaneously, ultimately leading to the full aluminum layer shear fracture. With the increase in testing temperature, the strength of aluminum decreases more significantly than that of copper and interface bonding. Eventually, the shear fracture mechanism is directly related to the difference between the interfacial bonding strength of the composites and the strength of aluminum.

## 4. Conclusions

The interfacial microstructure, interfacial shear strength, interfacial shear fracture morphology, and microstructure near the shear fracture of C18150Cu/1060Al/C18150Cu trilayered composite at different temperatures fabricated by high-temperature oxygen-free hot rolling technology were systematically investigated to reveal the shear fracture mechanism. The main conclusions are as follows:

(1) Al_2_Cu, AlCu, and Al_4_Cu_9_ intermetallics are formed at the interface of the prepared C18150Cu/1060Al/C18150Cu trilayered composite fabricated by high-temperature oxygen-free rolling, establishing metallurgical bonding, in which Al_2_Cu is preferentially formed. The interface bonding is achieved through both metallurgical and mechanical interactions, along with the copper and aluminum forming a mechanical interlock.
(2) The interfacial shear strength of the composites decreases with increase in testing temperature. However, when the testing temperature is less than 150 °C, the interfacial shear strength decreases slightly, and it has excellent thermal stability in this temperature range. At temperatures above 150 °C, the shear strength drops sharply, from 56.8 MPa at room temperature to 20.9 MPa at 350 °C.
(3) The intermetallics are mainly distributed on the aluminum surface of the shear fracture, with minimal presence on the copper surface of the shear fracture. The interfacial bonding strength between the intermetallics and the aluminum is higher than that between the intermetallics and the copper.
(4) At room temperature, the shear fracture mainly occurs at the interface between the copper and IMCs, with minimal aluminum presence on the copper surface at the shear fracture. The amount and size of the attached aluminum to the copper surface slowly grows as the temperature increases, and the curled aluminum that is not fully peeled off becomes evident. At 300 °C, signs of aluminum layer fracture appear, and at 350 °C, the aluminum layer shear fracture comprises approximately 75%, with the remaining 25% being interface fracture. As the temperature rises, the shear fracture mechanism transitions from interface shear fracture to aluminum layer shear fracture.

## Figures and Tables

**Table 1:** The chemical composition of 1060 aluminum alloy strip in wt.% [31].

Elements	Si	Fe	Mn	Zn	Cu	Mg	Al
Content	0.2	0.25	0.02	0.04	0.05	0.03	bal.

**Table 2:** The chemical composition of C18150 copper alloy strip in wt.%.

Elements	Cr	Zr	Zn	Al	Ni	Si	Cu
Content	0.72	0.102	0.042	0.023	0.015	0.0079	bal.

**Table 3:** The EDS results of IMCs in the C18150Cu/1060Al/C18150Cu trilayered composite.

Point	Element	Atomic (%)	Intermetallics
A	Al	66.86	Al_2_Cu
	Cu	33.14	
B	Al	32.77	Al_4_Cu_9_
	Cu	67.23	
C	Al	48.12	AlCu
	Cu	51.88	
D	Al	65.2	Al_2_Cu
	Cu	34.8	

**Table 4:** The interfacial shear strength of C18150Cu/1060Al/C18150Cu trilayered composite.

Temperature/°C	Interfacial Shear Strength/MPa
25	56.83
100	53.5
150	55.47
200	41.85
250	38.5
300	27.23
350	20.95

## Data Availability

The original contributions presented in this study are included in the article. Further inquiries can be directed to the corresponding authors.

## References

[1] Xing B.H., Huang T., Song K.X., Xu L.J., Xiang N., Chen X.W., Chen F.X. (2022). Effect of electric current on formability and microstructure evolution of Cu/Al laminated composite. J. Mater. Res. Technol..

[2] Song H., Hao W.X., Mu X.W., Han T.Z., Che C.J., Geng G.H. (2020). Effect of Pulse Current-Assisted Rolling on the Interface Bonding Strength and Microstructure of Cu/Al Laminated Composite. Metals.

[3] Ren Z.K., Gao X.Y., Hou J.X., Huang Z.Q., Wang T., Huang Q.X., Liu X. (2024). Achieving excellent strength-elongation synergy in corrugated Cu/Al laminated composites by flat finish roll bonding. J. Alloys Compd..

[4] Wang L., Liu J., Kong C., Pesin A., Zhilyaev A.P., Yu H.L. (2020). Sandwich-like Cu/Al/Cu composites fabricated by cryorolling. Adv. Eng. Mater..

[5] Gao H.T., Li J., Lei G., Song L.L., Kong C., Yu H.L. (2022). High Strength and Thermal Stability of Multilayered Cu/Al Composites Fabricated Through Accumulative Roll Bonding and Cryorolling. Metall. Mater. Trans. A.

[6] Li X., Du Q.Y., Sun M.H., Liu X.T., Zhao C. (2022). Study on the mechanism of strengthening the bonding strength of Cu/Al composite strip by cross shear behaviour from vibration cast-rolling process. Int. J. Adv. Manuf. Technol..

[7] Wang L., Du Q.L., Cui X.H., Zhao X., Yu H.L. (2019). Enhanced mechanical properties of lamellar Cu/Al composites processed via high-temperature accumulative roll bonding. Trans. Nonferrous Met. Soc. China.

[8] Bian G.B., Zhang T.T., Ran M.T., Zhang K.J., Zeng M., Zhou L.D., Pei L.X., Wang W.X. (2024). Interfacial microstructure and fracture mechanism of Cu/Al composite plate jointing process assisted by pulsed current-melted Al spheres: Finite element analysis and experimental studies. J. Alloys Compd..

[9] Wang T., Li S., Ren Z.K., Han J.C., Huang Q.X. (2019). A novel approach for preparing Cu/Al laminated composite based on corrugated roll. Mater. Lett..

[10] Radim K., Adéla M., Lenka K., František F. (2015). Fabrication and characterization of cold-swaged multilayered Al-Cu clad composites. Mater. Des..

[11] Radim K., Lenka K., Adéla M., Michal Š. (2017). Improvement of mechanical and electrical properties of rotary swaged Al-Cu clad composites. Mater. Des..

[12] Kuňcická L., Kocich R., Kǎcor P., Jambor M., Marek M. (2022). Characterising Correlations between Electric Conductivity and Structural Features in Rotary Swaged Al/Cu Laminated Conductors. Materials.

[13] Kuňcická L., Kocich R. (2023). Effect of Stacking Sequence on Mechanical Properties and Microstructural Features within Al/Cu Laminates. Materials.

[14] Thaneshan S., Shahin K., Saden H. (2013). Spiral extrusion of aluminum/copper composite for future manufacturing of hybrid rods: A study of bond strength and interfacial characteristics. J. Alloys Compd..

[15] Piotr B., Aleksandra B., Barbara R.B., Bogusława A.C., Dai J.Y., Małgorzata L., Terence G.L. (2020). Superior strength of tri-layered Al-Cu-Al nano-composites processed by high-pressure torsion. J. Alloys Compd..

[16] Sova A., Doubenskaia M., Trofimov E., Samodurova M. (2022). Deposition of High-Entropy Alloy Coating by Cold Spray Combined with Laser Melting: Feasibility Tests. J. Therm. Spray Technol..

[17] Moslem T., Morteza A., Sebastian L. (2024). Characterizing of a unique Al/Cu FGMMC fabricated via the ARB-CRB process followed by annealing. J. Alloys Compd..

[18] Huang X.Q., Lu Z.Y., Cai M.H., Peter D.H. (2020). Strain Hardening Behavior of Laminate Structure with Stable bcc/fcc Bimetal Interfaces. Front. Mater..

[19] Yoji M., Takuya Y., Toshiyuki F. (2023). Plastic instability criterion based on new necking parameters for Cu-Al, Cu-A5052, and Cu–A5083 roll-bonded laminated metal composites fabricated without post-annealing. J. Mater. Process. Technol..

[20] Huang T., Xing B.H., Song K.X., Xu L.J., Yan S.L., Xiang N., Guo J.Q., Zhang X.B., Huang L. (2023). Thermal and non-thermal effects of Cu/Al laminated composite during electrically assisted tension. Mater. Sci. Eng. A.

[21] Zhou Y.Y., Jiang F.C., Wang Z.Q., Chen J.Y. (2023). Microstructure characteristics and mechanical properties of Cu/Al laminated metal composites fabricated by electropulsing assisted ultrasonic additive manufacturing. J. Mater. Process. Technol..

[22] Lin H.R., Tian Y.Z., Sun S.J., Zhang Z.F. (2021). Microstructural Evolution and Mechanical Properties of Laminated CuAl Composites Processed by Accumulative Roll-Bonding and Annealing. Acta Metall. Sin. Engl. Lett..

[23] Tian S.K., Zhao F., Wu G.L., Liu M., Godfrey A., Xie J.X., Liu X.H. (2024). Dowel-like morphology of Cu_2_Al_3_ enhances shear strength of interfacial layers in Cu-Al composites. Acta Mater..

[24] Pintore M., Wölck J., Mittler T., Greß T., Volk W., Tonn B. (2020). Composite Casting and Characterization of Cu-Al Bilayer Compounds. Int. J. Met..

[25] Zhang Y.C., Wang A.Q., Liang T.T., Zhang J.H., Mao Z.P., Yang D., Xie J.P., Zhang H.J. (2024). Study on strengthening and toughening mechanisms of Cu/Al composites dominated by interface layer. Mater. Charact..

[26] Ji J.L., Wang A.Q., Liu P., Yang D., Zhu H.Y., Zhang Y.C., Wang J., Xie J.P. (2024). Microstructure and properties of C18150 Cu/8011 Al/C18150 Cu laminated composites. J. Mater. Res. Technol..

[27] Shen Z., Lin Z.Z., Shi P.J., Zhu J.L., Zheng T.X., Ding B., Guo Y.F., Zhong Y.B. (2022). Enhanced electrical, mechanical and tribological properties of Cu-Cr-Zr alloys by continuous extrusion forming and subsequent aging treatment. J. Mater. Sci. Technol..

[28] Xia C.D., Pang Y., Jia Y.L., Ni C.Y., Sheng X.F., Wang S.F., Jiang X.Y., Zhou Z.Z. (2022). Orientation relationships between precipitates and matrix and their crystallographic transformation in a Cu-Cr-Zr alloy. Mater. Sci. Eng. A.

[29] Cao X.Y., Niu W.Q., Pei W.L., Huang Z.Q., Wang T., Ma L.F. (2023). Deformation behavior and bonding properties of Cu/Al laminated composite plate by corrugated cold roll bonding. J. Mater. Res. Technol..

[30] Wang T., Gao X.Y., Zhang Z.X., Ren Z.K., Qi Y.Y., Zhao J.W. (2021). Interfacial bonding mechanism of Cu/Al composite plate produced by corrugated cold roll bonding. Rare Met..

[31] Cai H.S., Wang Q.D., Zhang N.N., Ebrahimi M., Zhao Y.C., Liu L., Guo F. (2024). Shear behavior of Cu/Al/Cu trilayered composites prepared by high-temperature oxygen-free rolling. J. Alloys Compd..

[32] Fu X., Wang R., Zhu Q.F., Wang P., Zuo Y.B. (2020). Effect of Annealing on the Interface and Mechanical Properties of Cu-Al-Cu Laminated Composite Prepared with Cold Rolling. Materials.

[33] Li L.X., Wang Y.H., Sun M.H., Du F.S. (2020). First principles study on interface optimization mechanism of Cu-Al composite plate and strip by vibration cast-rolling technology. Compos. Interfaces.

[34] Wang J., Zhao F., Wang R., Liu X.H. (2023). Preparation and properties of copper-aluminum composite strips and foils by horizontal continuous composite casting and rolling. J. Mater. Process. Technol..

[35] Zhang Y.B., Fu Y., Jie J.C., Wu L., Svynarenko K., Guo Q.T., Li T.J., Wang T.M. (2017). Characteristics of copper-clad aluminum rods prepared by horizontal continuous casting. Met. Mater. Int..

[36] Liu G.P., Wang Q.D., Zhang L., Ye B., Jiang H.Y., Ding W.J. (2018). Effect of Cooling Rate on the Microstructure and Mechanical Properties of Cu/Al Bimetal Fabricated by Compound Casting. Metall. Mater. Trans. A.

[37] Liu G.P., Wang Q.D., Zhang L., Ye B., Jiang H.Y., Ding W.J. (2019). Effects of Melt-to-Solid Volume Ratio and Pouring Temperature on Microstructures and Mechanical Properties of Cu/Al Bimetals in Compound Casting Process. Metall. Mater. Trans. A.

[38] Wei Y.N., Li H., Sun F., Zou J.T. (2019). The Interfacial Characterization and Performance of Cu/Al-Conductive Heads Processed by Explosion Welding, Cold Pressure Welding, and Solid-Liquid Casting. Metals.

[39] Liu T., Wang Q.D., Sui Y.D., Wang Q.G., Ding W.J. (2016). An investigation into interface formation and mechanical properties of aluminum–copper bimetal by squeeze casting. Mater. Des..

